# Effect of Genetic and Environmental Factors on Twin Pregnancy in Primiparous Dairy Cows

**DOI:** 10.3390/ani13122008

**Published:** 2023-06-16

**Authors:** Fernando López-Gatius, Irina Garcia-Ispierto, Sergi Ganau, Robert Wijma, Daniel J. Weigel, Fernando A. Di Croce

**Affiliations:** 1 Agrotecnio Centre, 25198 Lleida, Spain; 2 Transfer in Bovine Reproduction SLu, 22300 Barbastro, Spain; 3 Department of Animal Science, University of Lleida, 25198 Lleida, Spain; 4 Granja Sant Josep, La Melusa, 22549 Tamarite, Spain; 5 Zoetis Inc., 333 Portage Street, Kalamazoo, MI 49007, USA

**Keywords:** breeding strategy, dark–light cycles, dead co-twin, early fetal loss, selection index, spontaneous twin reduction, standardized transmitting ability

## Abstract

**Simple Summary:**

The biological and economic impacts of pregnancy loss in dairy herds are well recognized. Twin pregnancies compromise the health and wellbeing of cattle. The use of genomic testing for production traits and fitness traits to select replacement heifers is increasing in commercial dairy farms. Recently, a genomic prediction for twin pregnancies has been developed. The abortion of twin calves or parturition in the delivery of twins have been associated with the twin pregnancy trait (TWIN). However, the incidence of twins at abortion or at parturition are not a true reflection of twin pregnancy. A large number of pregnancy losses and twin reduction during the early fetal period are beyond clinical control. The rate of twin reduction is much higher than the rates of abortion and twin deliveries. The aim of this study was to evaluate factors affecting the incidence of twin pregnancies in high-producing primiparous dairy cows, with special emphasis placed on the genomic prediction values for twin pregnancy. Our study population of primiparous cows proved valuable in identifying factors other than genomic predictive values influencing the twin pregnancy rate. The photoperiod, milk production, and estrus synchronization protocol for fixed-time artificial insemination were found to significantly influence the rates of twin pregnancy.

**Abstract:**

Twin pregnancies are highly undesirable in dairy cattle; they compromise the health and wellbeing of a cow and dramatically impair the farm economy. Recently, a genomic prediction for twin pregnancies has been developed. The objective of this study was to assess environmental and management risk factors affecting the incidence of twin pregnancies in high-producing dairy cows in their first lactation, with a special emphasis placed on the genomic prediction values of twin pregnancy. Our study population of primiparous cows proved valuable in identifying factors other than genomic predictive values that influence twin pregnancy rates. The odds ratio for twin pregnancies was 0.85 (*p* < 0.0001) for each unit of a prediction value increase, 3.5 (*p* = 0.023) for cows becoming pregnant during the negative photoperiod, and 0.33 (*p* = 0.016) for cows producing ≥42 kg of milk at AI, compared with the remaining cows who produced <42 kg of milk. As a general conclusion, the practical implication of our findings is that genomic prediction values can identify the risk of twin pregnancy at a herd level. Given the cumulative effect of genomic selection, selecting animals with a reduced genetic risk of twin pregnancies can contribute to reducing the incidence of twin pregnancies in dairy herds.

## 1. Introduction

Twin pregnancies are highly undesirable in dairy cattle; they compromise the health and wellbeing of a cow. Compared with cows carrying singletons, cows carrying twins have a risk of pregnancy loss that is seven times higher during the first 90 days of gestation [1,2], a higher than 20% incidence of abortions during the second and third trimester of gestation [3], and a higher risk of perinatal mortality and postpartum reproductive disorders after twin delivery [4,5,6,7]. As a consequence, twin pregnancy reduces the lifespan of a cow (by 200 days) [4,8,9], and its incidence dramatically impairs farm economy [10,11,12,13]. A clear example is the economic impact per twin pregnancy, which is estimated to be between USD 97 and USD 225, depending on whether it is a unilateral or bilateral twin gestation, the age of the cow and the lactation period when gestation occurs [12]. Twins are more frequent in older cows and can reach about 30% of all pregnancies in cows with three or more lactations [14,15]. Depending on the location of the embryos, twin pregnancies are classified as unilateral, with both embryos in the same uterine horn (56%), or bilateral, with an embryo in each uterine horn (44%) [14,15]. Management strategies to prevent twin pregnancies, such as the draining of follicles of pre-ovulatory size (except the dominant one at AI), the transfer of a single embryo to non-inseminated cows, or induced twin reduction at pregnancy diagnosis, are the procedures available today [16,17].

The use of genomic testing for production traits and fitness traits to select replacement heifers is increasing in commercial dairy farms. In fact, current selection indices include both production and lifetime profitability traits [18,19,20,21]. A multi-trait selection index, which includes cow and calf wellness, milk production, fertility, abortion, and longevity, has been validated in commercial dairy herds to select healthier cows with longer lifespans [22,23,24]. Recently, a genomic prediction for twin pregnancies has been developed based on twin calving or abortion [25]. However, the incidence of twin pregnancies is greater than that observed in twin births or abortions. The incidence of losses between 28 and 60 days of gestation is greater in cows carrying twins [1,2] and can exceed 50% during the warm period of the year [15,26]. Moreover, natural twin reduction (the death of a single twin) usually occurs following a positive pregnancy diagnosis, with an incidence of cows maintaining gestation after experiencing a twin reduction up to 28% on Day 60 of gestation [1]. Both late embryonic or early fetal loss and spontaneous twin reduction often escape clinical diagnosis. Therefore, there is a great difference between the rate of twin pregnancies that can be observed at pregnancy diagnosis (i.e., 30–40 days after AI) and the subsequent rates of twin calvings or abortions. The diagnosis of twins towards the end of the embryonic period, i.e., days 30 to 40 of gestation, is the most appropriate target when investigating factors affecting twin pregnancy rates [1,14,15].

Lactation number, photoperiod, season, and management related to estrus synchronization protocols for fixed-time artificial insemination (FTAI) were found to influence the twin pregnancy rate in an extensive previous study [27]. As it was demonstrated that genetic correlations between TWIN and other traits were low [25], it should be expected that genomic prediction values for twin pregnancy can be used as a variable to study factors influencing twin pregnancy rates. The objective of this retrospective cohort study was to assess environmental, and management risk factors affecting the incidence of twin pregnancies in high-producing dairy cows, with a special emphasis on genomic prediction values in twin pregnancy. Our hypotheses were that twin pregnancy rates would be influenced by genetic value, photoperiod, and milk production.

## 2. Materials and Methods

### 2.1. Cows and Herd Management

The data used for this study were collected from a Holstein dairy herd in northeastern Spain (latitude 41.13 N, longitude −2.4 E). During the study period (March 2022 to February 2023), the mean number of lactating cows in the herd was 3580 and mean annual milk production was 15,695 kg per lactating cow. Cows were milked three times daily, and the mean annual culling rate during the study period was 30%. Cows were grouped according to age (primiparous cows, secundiparous cows, or cows in their third lactation or higher) and fed complete rations. Walking activity values were recorded at the milking parlor (three times daily) and automatically analyzed using a herd management computer program (AfiFarm System; Afikim. Israel). Herd management included the use of fans and water sprinklers. Fans were placed throughout cubicle and feeding areas, whereas water sprinklers were set up in the feeding area with water spray directed towards the cows. Fans and water sprinklers were automatically activated when temperatures reached approximately 23 °C and 25 °C, respectively. 

Only primiparous cows delivering singletons experiencing their first postpartum pregnancy and with complete information from insemination to pregnancy diagnosis were included in the study. Only healthy cows were included in the study, as indicated by a body condition score of 2–3.5 on a scale of 1 to 5 [28]. These cows produced more than 30 kg of milk per day at the time of AI and were free of clinical signs of disease during the study period (days −20 to 34 of insemination). All animals were reared within the herd.

### 2.2. Artificial Insemination and Ultrasound Exams

All cows were artificially inseminated, and the herd was maintained on a weekly reproductive health program as described elsewhere [14,15]. The voluntary waiting period for primiparous cows was 85 days. Cows showing spontaneous estrus after this time were inseminated. Cows reaching 80 days old who produced milk with no estrous signs for at least 21 days were synchronized for FTAI using the G-6-G protocol for cows with a functional corpus luteum (CL), a progesterone(P4)-based protocol for cows with no luteal structures, or with an apparently nonfunctional CL. The presence of one or more CL of at least 15 mm in diameter (defined as a functional CL) was assessed using ultrasonography. The size of the CL was taken from the mean of two measurements at the longest and widest axes. A lack of high pixel intensity associated with a young CL [29,30] was used as reference to assess CL functionality. As the presence of a central cavity is not functionally important [31,32,33], cavity CL were measured just like non-cavity CL.

Cows with a functional CL received cloprosternol (500 μg im; Cyclix bovino, Virbac España, Esplugues de Llobregat, Barcelona, Spain); 2 d, GnRH (100 μg im; Cystoreline, CEVA Salud Animal, Barcelona, Spain); 6 d, GnRH; 7 d, cloprostenol; 56 h, GnRH; 16–20 h, FTAI. Cows in absence of a functional CL were treated with a controlled intravaginal progesterone-releasing device (CIDR) (containing 1.38 g of progesterone; Zoetis Spain SL, Alcobendas, Madrid, Spain) plus GnRH upon CIDR insertion. The CIDR was left in place for 5 d, and these animals were also given cloprostenol upon CIDR removal. Then 24 h and 36 h later, the cows received a second cloprostenol dose and a second GnRH dose, respectively, and FTAI was found 68–72 h after CIDR removal. Both are established protocols in routine farm reproductive management.

Pregnancy was diagnosed using transrectal ultrasonography 31 ± 3 days post-AI via a portable B-mode ultrasound scanner equipped with a 5–10 MHz transducer (E.I. Medical IBEX LITE, E.I. Medical Imaging, Loveland, CO, USA). Each ovary was scanned in several planes by moving the transducer along its surface to identify luteal structures, and the size, number and location of CL were recorded. Scanning was then performed along the dorso/lateral surface of each uterine horn. The presence of twins was established via the observation of two embryos in different positions within one uterine horn on two screen scans, two embryos simultaneously present on the screen (unilateral twin pregnancy), or one embryo in each uterine horn (bilateral twin pregnancy). The final study population comprised 775 cows: 29 carrying twins (11 unilateral plus 18 bilateral twins) and 746 carrying singletons. Because twin reduction by amnion rupture is routinely performed in this herd at pregnancy diagnosis (31 ± 3 days post-AI) in bilateral twin situations [16,17], the number of fetuses was only confirmed in cows carrying unilateral twins or singletons by ultrasound 49 to 55 days post-AI. All gynecological exams and pregnancy diagnoses were performed by the same operator.

### 2.3. Evaluation of Genetic Merit

Ear tissue samples were collected from all animals between six and ten months of age and submitted for genomic testing using the Clarifide Plus evaluation (Zoetis Genetics, Kalamazoo, MI, USA). This is part of the farm’s routine and complies with the current legislation regarding animal welfare. The Clarifide Plus evaluation uses the Council of Dairy Cattle Breeding (CDCB) predictions, as well as exclusive health and fertility traits [21,22,24,25] and profitability indexes [23]. Within these fertility traits, Clarifide Plus provides a genomic prediction for risk of twin pregnancies which has been previously described by McGovern et al. [25]. Genomic predictions for twin pregnancies are expressed as standardized transmitting abilities (STA) with a mean of 100 and standard deviation (S.D.) of 5. Greater genomic STA values represent a lesser risk of having a twin pregnancy.

### 2.4. Data Collection and Statistical Analyses

The twin pregnancy rate was defined as the percentage of pregnant cows carrying twins at 31 ± 3 days post-AI. The following data were recorded in each animal: parturition and AI dates; estrus at AI (spontaneous estrus, G-6-G synchronization or P4-based synchronization); lactation days at AI; milk production at AI (mean production in the seven days before AI) (low producers <42 kg vs. high producers ≥42 kg); AI number; genomic prediction values for twin pregnancy; pregnancy 28–34 days post-AI; and presence of twins in pregnant cows. Since the manifestation of estrus often leads to a reduction in milk production, the mean of the seven days prior to estrus was obtained and used as milk production data at the time of AI. The threshold for milk production was set as the mean value of production recorded. AI dates were used to assess the effects of photoperiod (positive or negative) and season on the incidence of twin pregnancies. The positive photoperiod extends from December 22 to June 21. In the region where the farm is located, there are only two clearly distinguishable climatic periods: warm (May to September) and cool (October to April) [26,27]. During the cool period, temperatures <0 °C were recorded on 29 days and temperatures >25 °C on 5 days. During the warm period, temperatures >25 °C were recorded on 101 days.

The software package PASW Statistics for Windows Version 18.0 (SPSS Inc., Chicago, IL, USA) was used for data processing. Significance was set at *p* < 0.05. Variables are expressed as the mean ± S.D. A binary logistic regression analysis was performed using twin pregnancy as the dependent variable. The factors entered in the model were genomic prediction values and lactation days as continuous variables; season of AI (warm), photoperiod (positive), pregnancy at first AI and milk production (high production) as dichotomous variables (where “1” denotes presence and “0” absence); and estrus at AI (spontaneous estrus, G-6-G synchronization or P4-based synchronization) as a class variable. Possible interactions between milk production and season or photoperiod were also investigated. Regression analyses were conducted according to the method of Hosmer and Lemeshow [34]. Essentially, this method consists of five steps as follows: preliminary screening of all variables for univariate associations; construction of a full model using all the significant variables arising from the univariate analysis; stepwise removal of non-significant variables from the full model and comparison of the reduced model with the previous model for model fit and confounding; evaluation of plausible interactions among variables; and assessment of model fit using Hosmer–Lemeshow statistics. Variables with univariate associations with *p* values < 0.25 were included in the initial model. Modelling was continued until all the main effects or interaction terms were significant according to the Wald statistic at *p* < 0.05 and remained in the model.

## 3. Results

Of the 775 cows, 29 (3.7%) carried twins, 330 (42.6%) became pregnant during the warm period, 268 (34.6%) during the positive photoperiod, and 458 (59.1%) at the first AI. At least one CL ipsilateral to the embryo was observed in all twin pregnancies. Triplets or quadruplets were not recorded. Milk production and lactation days at the time of AI, AI number, as well as genomic STA values were 42 ± 5.8 (32–59) kg, 114 ± 42 (85–330) days, 1.8 ± 1.3 (1–9) AIs, and 100 ± 4.5 (82–110) units, respectively (mean ± SD; ranges between parentheses). Of the 330 pregnancies registered during the warm period, 9 (2.7%) were of twins. Of the 458 cows becoming pregnant at the first AI, 15 (3.3%) carried twins and had a mean of lactation days at the time of AI of 92 ± 5.4 (85–114) days.

Table 1 summarizes the twin pregnancy rate, odds ratio and 95% confidence interval for all cows. The final model included the effect of genomic prediction values, photoperiod, milk production and reproductive treatment. The warm period of the year, lactation days and pregnancy at first AI were not significant and not included in the model. Season–milk production, photoperiod–milk production, reproductive treatment–season, reproductive treatment–photoperiod or reproductive treatment–milk production interactions were not found. The odds ratio for twin pregnancy was 0.85 (*p* < 0.0001) for each unit increase in genomic STA, 3.5 (*p* = 0.023) for cows becoming pregnant during the negative photoperiod, and 0.33 (*p* = 0.016) for cows producing ≥42 kg of milk at AI, compared to cows producing <42 kg of milk. Using spontaneous estrus as reference, the odds ratio for twin pregnancy was 3.1 (*p* = 0.019) for cows receiving the G-6-G protocol. The influence of the P4-based protocol was not significant (Table 1).

As cows were inseminated over a long period of days in lactation (between 85 and 330 days) and received between 1 and 9 AI, a further binary logistic regression analysis was performed including only cows becoming pregnant at the first AI (*n* = 458). Of the 458 cows, 43 (9.4%) received the G-6-G protocol and 46 (10%) the p4-based protocol. Table 2 summarizes the twin pregnancy rate, odds ratio, and 95% confidence interval for cows becoming pregnant at the first AI. The final model included the effects of genomic prediction values, photoperiod, and milk production. The influence of reproductive treatment and season were not significant, and interactions were not found. As Table 2 shows a slightly worse fit and significances compared to Table 1, we mainly discuss the data shown in Table 1.

## 4. Discussion

The incidence of twin pregnancies continues to rise in parallel with increased milk production [1,4,25,35,36]. As early as 1924, Hunt wrote: “There is a definite relationship between high fertility and high milk production… This relationship seems to hold true when high fertility is evidenced in the production of twins” [37]. However, younger females have remained over time the lowest risk group for producing twins. Primiparous cows rarely reach a twinning rate of 2% [8,38]. This is expected, given that maturity of the reproductive tract and the highest milk production is achieved in multiparous cows, which experience a higher incidence of multiple pregnancies [4,8,35,36,38]. With a 3.7% (29/775) rate of twin pregnancies, our study population of primiparous cows proved valuable in identifying factors other than genomic predictive values that influence twin pregnancy rates. Factors such as the photoperiod, milk production and estrus synchronization protocol for FTAI were found to significantly influence the rate of twin pregnancy.

Our results are in agreement with McGovern et al. [25], who observed a reduction in twin pregnancies in those cows with greater genomic STA values. Interestingly, in contrast to what we observed in the present study, the authors observed an interaction between genomic predictions and herd reproductive management, with increased risk of twin pregnancy in those herds that relied more on heat detection. In the current study we observed an increase in the probability of twin pregnancy in cows synchronized with the G-6-G protocol. Nevertheless, given the reduced number of individuals in the synchronization groups, the overall low incidence of twin pregnancies, and the fact that only primiparous cows were included, more research is needed to further explore an effect of spontaneous estrus breeding on reducing twin pregnancies.

Regardless of genomic prediction values, the length of the photoperiod influenced the incidence of twin pregnancies by a factor of 3.5. Although cows are not strict season breeders, a negative photoperiod led to higher twin pregnancy rates (4.9%) compared to a positive photoperiod (1.5%), reinforcing previous results [27]. We tested a possible interaction between genomic prediction values and photoperiod on the twin pregnancy rate. A further binary logistic regression analysis was performed, including genomic prediction values as a dichotomous variable (low prediction values < 100 units vs. high prediction values ≥ 100 units). The outcome of the model (not presented) was practically the same as that presented in Table 1. However, the model was had a worse fit (*p* = 0.60) than the model with genomic prediction values as a continuous variable (*p* = 0.92), and a genomic prediction value–photoperiod interaction was not found. Therefore, we considered that the independent variable genomic prediction values, considered as a continuous variable, were a better predictor of twin pregnancy than as a dichotomous variable. 

The influence of a decreasing photoperiod length on the increased incidence of twin pregnancies may well originate from an ancient strategy in mammals, increasing the chances of parturition and numbers of offspring when feed availability is higher [39,40,41]. The fact that the warm period of the year had no effects on the twin pregnancy rate found in the present study reinforces this suggestion.

Heat stress is a great problem in animals and humans [42], especially in high-producing dairy cows [43,44,45]. Although the warm period of the year greatly impairs the management of twin pregnancies [15,26], the season had no effects on the incidence of twins in the present study. In a previous study performed between 2010 and 2011 in herds located in our geographical area, with a mean annual milk production around 11,500 kg per cow, both warm period and positive photoperiods were associated with a significant decrease in the incidence of twin pregnancies [27]. As the warm period of the year (April to September) includes part of negative and positive photoperiod, although interactions were not found, it is possible that the effects of one of the two factors masked the effects of the other in the referenced report [27]. In a subsequent experiment conducted on cows in their third lactation or higher in the same farm as the present study, with a mean annual milk production of 12,230 kg per cow from 2014 to 2018, the twin pregnancy rate was similar for both warm (26.5%) and cool (26.3%) periods [15]. Currently, the herd is producing 15,695 kg of milk per cow every year (present study), so it seems clear that management practices associated with high milk production and heat abatement mitigate the negative effects of heat stress on twin pregnancy rate. Therefore, the independence of season and photoperiod shown here indicates the strong effect of photoperiod on the incidence of twins.

The likelihood of twin pregnancy decreased in high-producing cows by a factor of 0.33 in this study of first-lactation cows. Although, in recent decades, high milk production has been considered the most important single contributors to increasing ovulations, and thus twinning rates [1,4,35,36], the percentage of twin pregnancies was 2.2% in high producers (≥42 kg per day), in contrast to the 4.8% recorded in cows with low production (<42 kg). This is not entirely new. In the previous study cited above [27], using univariate analyses, it was observed that cows carrying twins produced less milk than cows carrying singletons: 40.5 ± 9.3 kg vs. 41.7 ± 9.1 kg, respectively (*p* = 0.032). However, after adjusting for other factors in the logistic regression analysis, milk production was not a factor associated with the likelihood of twin pregnancy [27]. These findings question the hypothesis that high-producing dairy cows may be associated with double ovulation and a risk of twin pregnancies [46,47,48,49]. It is clear that multiple ovulations can lead to twin pregnancies. More than 90% of twins derive from two or more ovulations (i.e., dizygotic twins) [50,51]. However, the association between milk production and multiple ovulations has also been questioned in a recent review [52]. Improvements in management and nutrition practices, along with genetic progress, that have resulted in a steady increase in milk production [36,53,54] have probably reduced the incidence of pregnancy loss for twin pregnancies, therefore favoring an increased twinning rate in recent years. On the other hand, the fact that high-producing cows may show a lower twin pregnancy rate than their low-producing partners may have clinical implications. The ratio of the percentage of twins between low and high producers was 2.2 (4.8/2.2) in the present study. The question that arises is why the ratio of twins favors the twinning rate of low producers? On-farm management practices probably mitigated the effects of heat stress but not metabolic stress associated with high milk production. If this is the case, when examining the reproductive performance of a herd, the ratio of twins between low and high producers close to one could be a good indicator of cow wellbeing. This should be assessed in more extensive studies. Of course, in the case of low producers, another problem would be how to prevent multiple pregnancies.

## 5. Conclusions

As a general conclusion, the practical implication of our findings is that genomic prediction values can identify the risk of twin pregnancy at herd level. Given the cumulative effect of genomic selection, selecting for animals with reduced genetic risk of twin pregnancies can contribute to reducing the incidence of twin pregnancy in dairy herds. Furthermore, being able to identify those animals at greater risk may allow us to perform precision management to prevent twin pregnancies, such as the transfer of a single embryo, puncture and drainage of subordinate follicles at the time of AI, or the use of more sophisticated synchronization protocols.

## Figures and Tables

**Table 1:** Odds ratios of the twin pregnancy rate variables included in the final logistic regression model (*n* = 775 primiparous pregnant cows).

Factor	Class	*n*	Twin Pregnancy	Odds Ratio	95% Confidence Interval	*p*
ZTWINS ^(a)^	Continuous	29/775	3.7%	0.85	0.78–0.95	<0.0001
Photoperiod	Positive	4/268	1.5%	Reference		
	Negative	25/507	4.9%	3.5	1.2–10.5	0.023
Milk production (kg)	<42	22/458	4.8%	Reference		
	≥42	7/317	2.2%	0.33	0.13–0.81	0.016
Estrus ^(b)^	Spontaneous	16/578	2.8%	Reference		
	G-6-G protocol	7/102	6.9%	3.1	1.2–18.1	0.019
	P4 protocol	6/95	6.3%	1.8	0.6–5.3	0.22

Hosmer and Lemeshow goodness-of-fit test = 23.6; 3 df, *p* = 0.92. R^2^ Nagelkerke = 0.14. ^(a)^ Genomic prediction values for twin pregnancy. The lowest value (82 units) was used as the reference. ^(b)^ Type of estrus at pregnant insemination: Spontaneous; following a G-6-G protocol; following a progesterone(P4)-based protocol.

**Table 2:** Odds ratios of the twin pregnancy rate variables included in the final logistic regression model (*n* = 458 primiparous cows becoming pregnant at the first AI).

Factor	Class	*n*	Twin Pregnancy	Odds Ratio	95% Confidence Interval	*p*
ZTWINS ^(a)^	Continuous	15/458	3.3%	0.85	0.76–0.94	0.002
Photoperiod	Positive	2/185	1.1%	Reference		
	Negative	13/273	4.8%	4.4	1.01–20	0.048
Milk production (kg)	<42	14/306	4.6%	Reference		
	≥42	1/152	0.7%2	0.13	0.01–0.99	0.049

Hosmer and Lemeshow goodness-of-fit test = 20.6; 3 df, *p* = 0.90. R^2^ Nagelkerke = 0.17. ^(a)^ Genomic prediction values for twin pregnancy. The lowest value (82 units) was used as the reference.

## Data Availability

Data available on request due to restrictions (privacy). The data are not publicly available due to privacy and third-party agreements.
